# Development of Microsatellite Markers Derived from Expressed Sequence Tags of Polyporales for Genetic Diversity Analysis of Endangered *Polyporus umbellatus*

**DOI:** 10.1155/2015/941357

**Published:** 2015-06-03

**Authors:** Yuejin Zhang, Yuanyuan Chen, Ruihong Wang, Ailin Zeng, Michael K. Deyholos, Jia Shu, Hongbo Guo

**Affiliations:** ^1^State Key Laboratory of Crop Stress Biology for Arid Areas, College of Life Sciences, Northwest A&F University, Yangling 712100, China; ^2^Department of Biology, The University of British Columbia Okanagan, Kelowna, BC, Canada V1V 1V7

## Abstract

A large scale of EST sequences of Polyporales was screened in this investigation in order to identify EST-SSR markers for various applications. The distribution of EST sequences and SSRs in five families of Polyporales was analyzed, respectively. Mononucleotide was the most abundant type, followed by trinucleotide. Among five families, Ganodermataceae occupied the most SSR markers, followed by Coriolaceae. Functional prediction of SSR marker-containing EST sequences in* Ganoderma lucidum* obtained three main groups, namely, cellular component, biological process, and molecular function. Thirty EST-SSR primers were designed to evaluate the genetic diversity of 13 natural* Polyporus umbellatus* accessions. Twenty one EST-SSRs were polymorphic with average PIC value of 0.33 and transferability rate of 71%. These 13* P*.* umbellatus* accessions showed relatively high genetic diversity. The expected heterozygosity, Nei's gene diversity, and Shannon information index were 0.41, 0.39, and 0.57, respectively. Both UPGMA dendrogram and principal coordinate analysis (PCA) showed the same cluster result that divided the 13 accessions into three or four groups.

## 1. Introduction

*Polyporus umbellatus* (Pers.) Fries, a fungus that belongs to Polyporaceae, Polyporales, is widely distributed in China, Japan, Europe, and North America [1]. Its dried sclerotium has been used as a diuretic in traditional Chinese medicine for 2,500 years [2], which also exhibits many other pharmacological functions, that is,* in vivo* anticancer activity [3]. These potent pharmacological properties attract worldwide interest in developing this medicinal fungus. In the past, wild* P. umbellatus* was widely distributed in 13 provinces of China [4], but nowadays its distribution shrinks severely, which has been listed as an endangered species in China Red Book [5].

Molecular markers are frequently used to analyze genetic diversity for endangered species, and to date only sequence-related amplified polymorphism (SRAP) markers have been used to characterize* P. umbellatus* germplasm in our previous work [6]. Although that work provided valuable genetic information, the lack of codominant markers has hampered further its genetic evaluation. Fortunately, the expressed sequence tags (ESTs) in Polyporales provide valuable resources for DNA markers, because they may be functionally more informative than SSRs from unexpressed genome regions [7]. EST-derived SSR markers (EST-SSR) have several advantages over the other genomic DNA-based markers, such as detection of variation in coding sequences, a higher level of transferability to closely related species, and higher conservation than genomic SSRs [8].

In this investigation, a large scale of EST sequences (108,175) was screened and the aim of this study is (1) to characterize SSR markers from EST library of Polyporales and SSR-containing EST sequences for functional analysis and (2) to use newly developed SSR markers to evaluate genetic diversity and differentiation among 13 samples after validation. Our results can be used to develop more EST-SSR markers for those species in Polyporales and are very helpful for taxonomic, molecular breeding, and functional gene analysis.

## 2. Results

### 2.1. EST-SSR Markers in Polyporales

A total of 108,175 EST sequences from species in the order Polyporales were found in NCBI on September 11, 2013, including 58,902 in Coriolaceae, 48,721 in Ganodermataceae, 264 in Polyporaceae, 251 in Phanerochaetaceae, and 37 in Meruliaceae. From these, 97,134 nonredundant EST sequences were obtained with total length of 63.5 Mb. Among the nonredundant ESTs, 8,531 ESTs (8.78%) contained at least one SSR locus, with a total of identified 9,520 SSR loci, yielding an average frequency of occurrence 149.81/Mb. 484 EST sequences containing two or more SSR markers and 758 complex nucleotide repeats were found. As revealed by Table 1, all motif lengths ranging from mono- to hexanucleotide were detected with different repetitive frequency. Mononucleotide was the most abundant type, followed by trinucleotide; indeed, 94.5% of the identified repeats were either mono- or trinucleotide. In total, 55 different SSR motifs were found (Table 1). There were two identified mononucleotide motifs and A/T was the most repeat motif (88.02%). Among the pentanucleotide repeats, AAAAT/ATTTT was similarly overrepresented (84%).

The distribution of EST sequences and SSRs in five families of Polyporales was analyzed (Table 2). Most of the identified SSRs (7,188 loci; 75.5% of all SSRs) were found in the 48,721 ESTs from Ganodermataceae. A total of 2,287 SSR markers were identified in 58,902 EST sequences from Coriolaceae, representing 24.02% of all identified SSRs in Polyporales. In the ESTs sequences of Phanerochaetaceae and Polyporaceae, only 42 and 3 SSR markers were detected, respectively. No SSRs were found in ESTs from Meruliaceae.

There were 8,253 SSR markers (89.69% of total 9,520) with motifs repeating times more than 10, and 5,463 SSR sequences (57.38%) with length ranging from 10 to 19 bp. The numbers of SSR loci with length varying from 20 to 29 bp and longer than 30 bp were 2,678 and 1,379, respectively (Table 3). As far as their distribution in each family, 50% of SSRs in Ganodermataceae and Phanerochaetaceae were 20 bp or longer, whereas only 19.55% of SSRs were 20 bp or longer in Coriolaceae, No SSR sequence was found to be longer than 20 bp in Polyporaceae.

### 2.2. Analysis of SSR Sites in EST Sequences of Coriolaceae

From a total of 48,263 nonredundant EST sequences (30.6 Mb), 1,466 ESTs contained 2,287 SSR markers, in which 358 contained 667 complex markers (29.6%). The frequency of SSR marker occurrence in this family was 36/Mb.

All nucleotide repeats motifs from mono- to hexanucleotide were found in the EST sequences of Coriolaceae. Most of SSR loci (1,607, 70.27%) were mononucleotide repeats, followed by trinucleotide repeats (18.8%, Table 4). There were 1,622 SSR sites in this family with repetition more than 10 times, occupying the percentage of 70.92%. In addition, total 1,840 SSR sites (80.45%) with sequence length ranging from 10 to 19 bp were found, followed by 262 ones (11.46%) with 20–29 bp. The remaining 185 were the sites with sequence length longer than 30 bp.

### 2.3. Analysis of SSR Sites in the EST Sequences of Meruliaceae, Polyporaceae and Phanerochaetaceae

No SSR site was found in 37 EST sequences of Meruliaceae (total sequence length 7,961 bp). Only 3 SSR sites were detected among 264 EST sequences of Polyporaceae with sequence length 104,423 bp, namely, (T)10, (G)10, and (TC)6. The frequency of occurrence was 0.05/Mb with percentage of 1.14%.

A total of 243 EST sequences were obtained in Phanerochaetaceae with full length 141,652 bp, in which 37 were found to have SSR markers (15.22%). Among them, five sequences had complex markers with a total of 42, in which 41 was mononucleotide and dinucleotide (GA)_6_. The frequency of occurrence was 0.66/Mb.

### 2.4. Analysis of SSR Markers in the EST Sequences of Ganodermataceae

A total of 7,025 sequences (14.5%) containing 7,188 SSR markers were obtained from 48,331 EST sequences in Ganodermataceae. The percentage of SSR marker occurrence was 14.87% with average distance 4.54 kb between two markers. Out of 7,025 sequences, 121 had two or more SSR markers with 79 complex repeat motif types. The mononucleotide had the most repeats 6,530 (90.8%), followed by trinucleotide 386 with percentage of 5.37% (Table 5).

A total of 38 repeat motifs were found in EST-SSR sites of Ganodermataceae, among which hexanucleotide had the most repeat motifs (13), followed by trinucleotide (10). The tetranucleotide had 6 repeat motifs, namely, ACTG/AGTC, ACGC/CGTG, ACCT/AGGT, ACCG/CGGT, AATG/ATTC, and AACG/CGTT. The pentanucleotide contained three repeat motifs, that is, ATCCC, CGAAT, and TAAAA. As far as the frequency of occurrence, both mononucleotides A/T and C/G had the most repeat with 112 and 103 times, respectively, followed by AGC/CTG (85 times), ACG/CGT (65 times), AAG/CTT (63 times), and CCG/CGG (47 times). The other motifs, such as AAG/CTT, ACC/GGT, AGG/CCT, and ACCACG/CGTGGT, had repeat times more than 20. The thirteen hexanucleotide motifs appeared 69 times, among which the specific ACCACG/CGTGGT occupied 45 ones. The 22 motifs with the most repeats were analyzed in Table 6.

Those motifs with more than 10 repeats were characterized in EST sequences of this family, which occupied 91.65% (6,588 markers) of total 7,188 SSR markers. The percentage still would be 86% (6,187 markers), even if the repeat times increased to more than 13. When the repeat times were lower than 10 times, five (297 markers), six (163 markers), and seven (117 markers) were the most in turn.

There were 3,599 SSR markers (50.07%) containing repeat motifs with lengths 10–19 bp, followed by 1,880 markers (26.15%), whose sequence length of the repeat motif ranged from 20 to 29 bp. The number of markers that contained repeat motifs with sequence length more than 30 bp was 1186. In all these repeat motifs, the shortest sequence length was 12 bp (a trinucleotide repeat), while the longest was a GA repeat with 164 bp.

### 2.5. Functional Analysis of SSR Markers in EST Sequences of Ganodermataceae

**Figure 1:**
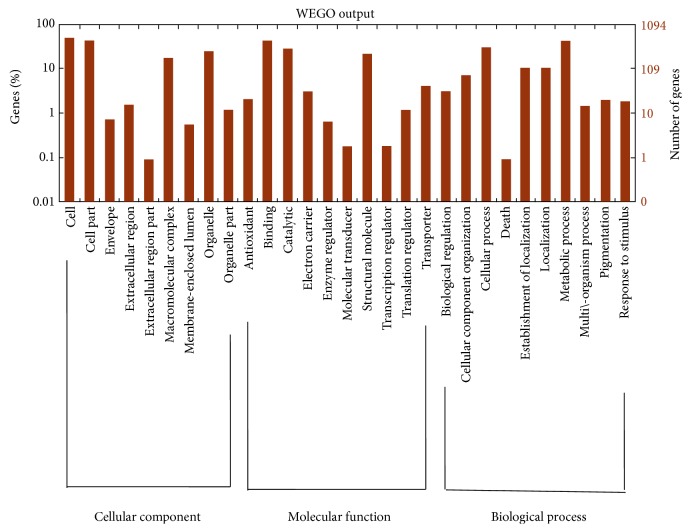
Functional analysis of 4370 SSR markers-containing EST sequences in Ganodermataceae by using gene ontology.

The Blast2GO tool was employed to predict the function of those EST sequences of Ganodermataceae that contained SSR markers. A total of 4,370 EST sequences were clustered into three groups and 33 subgroups based on gene ontology (GO) (Figure 1). Among them, cellular component had 12 subgroups, followed by biological process (11) and molecular function (10). The cellular component, biological process, and molecular function contained 1,954, 1,275, and 1,141 genes, respectively. Among 1,275 genes involved in biological process, the number of genes involved in metabolic process and cellular process was 452 and 359, respectively, followed by cellular location construction (222) and cellular location (111). For the molecular function, the subgroup mainly contained protein binding, catalytic activity, and structural molecule activity, including 469, 307, and 240 genes, respectively.

### 2.6. Genetic Diversity Analysis by Using Screened SSR Primers

All thirty primers were used to amplify thirteen* P. umbellatus* samples, among which 23 amplified PCR products. Except for PS01 and FP10, all primers amplified polymorphic bands among 13 samples and were used to calculate their polymorphism and genetic diversity. Both PS01 and FP10 only amplified monomorphic bands among these samples, but they would be valid if more samples were evaluated.

The 21 selected EST-SSR primers were used to amplify 13* P. umbellatus* samples, and the amplified 44 polymorphic bands ranging from 100 to 600 bp were recorded. The Shannon information index (*I*) ranged from 0.20 (GL09) to 0.92 (PS08) with an average of 0.57 (Table 7). The expected heterozygosity (*He*) varied from 0.14 (GL10) to 0.57 (PS08). Both* He* and* I* were the important estimate parameters of genetic diversity. In general, the level of polymorphism would be high if these two values were high [9]; however, the detailed value was determined by both the ability for a primer to identify polymorphism and the real polymorphism existing in samples [10].

The polymorphism information content (PIC) was a parameter that could evaluate the ability of a primer to identify polymorphism. The PIC value ranged from 0 to 1, in which the higher value would exhibit stronger ability. If PIC value was lower than 0.25, it showed that the primer only could provide less information. On the other hand, if the PIC value was higher than 0.5, it meant that the primer could provide more information [11]. In this investigation, the PIC value of 21 primers ranged from 0.09 to 0.48 based on their amplification on 13 samples. Both PS05 (0.46) and PS08 (0.48) yielded the largest values, while the lowest were found in GL09 (0.09), GL10 (0.12), and GL11 (0.13). Except for these three primers, the PIC values of the other 18 primers were all higher than 0.25, which could provide relative more information.

### 2.7. Analysis of Genetic Differentiation

Genetic similarity coefficient of 13* P. umbellatus* samples was calculated based on the methods provided by Nei [12]. The similarity coefficient ranged from 0.42 (strain 11 versus strain 2) to 0.80 (strain 1 versus strain 5) (Table 8). The results indicate that these strains have relatively higher differentiation.

**Figure 2:**
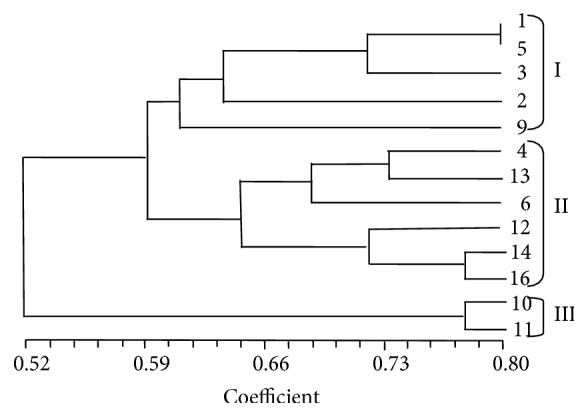
Dendrogram of 13* Polyporus umbellatus* strains constructed UPGMA method based on the amplification of 21 EST-SSR primers.

The UPGMA method was adopted to construct a dendrogram by using cluster analysis. If the threshold value 0.59 was defined, three clusters could be obtained (Figure 2). However, there was no obvious rule among them, that is, geographic correlation. In previous work, we discussed that different microenvironments might affect the composition of* Armillaria* species populations, which would form selection pressure on the genetic structure of local* P. umbellatus* strains [6].

**Figure 3:**
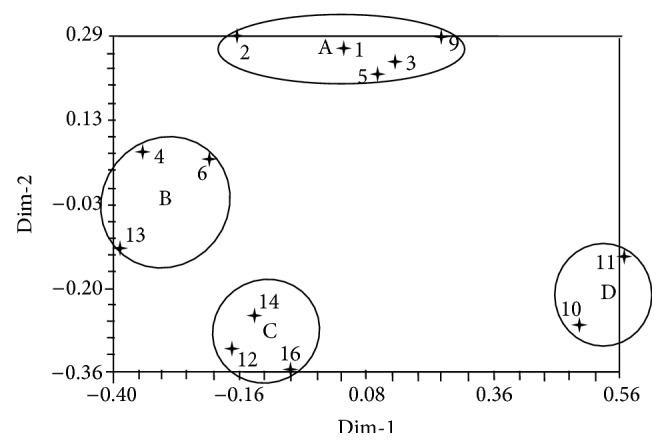
Principal ordinate analysis plot of the first two principal coordinates of 13 natural strains of* Polyporus umbellatus* based on the amplification of 21 selected EST-SSR primers.

The principal coordinate analysis (PCA) could exhibit the genetic distance between those clustered groups, which was different from UPGMA tree. If they were combined, they could provide more detailed information on the explanation of cluster results. As revealed by Figure 3, four clusters (from A to D) were obtained, which was in agreement with the three groups in UPGMA dendrogram. The only difference was that the Cluster II in dendrogram was divided into two groups (B and C) in PCA distribution.

## 3. Discussion

### 3.1. SSR Sites in EST Sequences

A total of 9,520 SSR markers were developed from 108,175 EST sequences of Polyporales with percentage of 9.8% and frequency of occurrence of 149.81/Mb. Among the four families that contained SSR markers, both the percentage and frequency of occurrence of markers differed. Both families of Phanerochaetaceae and Ganodermataceae had the highest percentage with 17.28 and 14.87%, respectively. However, a relatively low percentage was found in Coriolacae and Polyporaceae with 4.74 and 1.14%, respectively. The percentages of SSR-containing ESTs from Phanerochaetaceae and Ganodermataceae were higher than those in many previously described species, such as mushroom (2.99%, [13]), cotton (6.0%, [14]), hot pepper (8.44%, [15]), maize (7.25%, [16]), lily (5.98%, [17]), and sugarcane (4.7%, [18]). However, the difference of frequency of SSR-containing ESTs is partially dependent on the searching parameters. For an example, 12, 15, and 18 bp can be used as the searching length, but they will produce different results [19]. In this investigation, only 1.14% of the SSR sites were identified in EST sequences of Polyporaceae and nothing in Meruliaceae, which is attributed in part to the low number of available EST sequences in both families. In addition, the prefiltering of sequences, such as removing shorter sequences with length shorter than 100 bp and repetitive and redundant sequences, also will affect the percentage and frequency of occurrence. If the original EST sequences were not prefiltered, it could result in the repetitive development of SSR sites [19].

The di- and trinucleotide repeats are the main SSR-site type in most of plants that are derived from EST sequences, such as kale [20], lychee [21], and sesame [22], though the motifs may be different in different species. For the fungus Polyporales in this paper, some minor differences were noted in comparison with typical plant species. The mononucleotide repeat in the Polyporales was the main type (85.92% of all SSRs, frequency of 8.42% among ESTs, Table 1), while both di- and trinucleotide repeats only represented 2.73% and 8.57% of all identified SSRs, respectively (0.84 and 0.27% of ESTs, resp., Table 1). The A/T was the main repeat motif in mononucleotide, occupying 7.41% of total repeat motifs. This phenomenon existed in each family except for Coriolaceae, in which the percentage of G/C was slightly higher than A/T. The situation in which both mono- and trinucleotide repeats are the main type also exists in EST-SSR sites of castor [23] and tomato [24]. Notably, the abundant dinucleotide repeat motif AG/CT in many plant species was the same as it in Polyporales, though the percentage may be different. However, the abundant trinucleotide repeat motifs were different between plants and fungus. Surprisingly, the penta- and hexanucleotide repeat motif showed obvious preference. The AAAAT/ATTTT and ACCACG/CGTGGT motifs occupied 84.04 and 24.41% of penta- and hexanucleotide repeats, respectively. This motif preference is not reported in plant till now. In fact, both penta- and hexanucleotide repeats are not easy to find in plants, and their functions are unknown.

Most SSRs polymorphisms are thought to originate from errors during replication. In SSR sites, longer repeat sequences tend to have higher rates of polymorphism. Some researchers have reported that polymorphism will decrease if the length of repeat sequence is shorter than 20 bp, but it will increase when the sequences are longer than 20 bp [25, 26]. As far as the repeat number, Dreisigacker et al. [27] found that the polymorphism of SSR sites with fewer repeat times was higher than that with more repetitions. However, Zhan et al. [28] proposed a range for repeat times that was obtained from statistical data, and thought the polymorphism would be higher if the range was wider. In this paper, there are 8,253 EST sequences that had nucleotide repetition more than 10 times, which occupied 89.69% of total 9,520 in Polyporales. At the same time, the length of repeat sequences was 20 bp or more, occupying 42.62% of total. Therefore, the SSR sites derived from EST sequences of Polyporales should have higher polymorphism and bigger developmental potential.

### 3.2. Transferability of EST-SSR Primers and Analysis of Genetic Diversity

With the drastic increase of EST sequences in the NCBI database, it is appealing to use them to identify SSR loci for species of interest.* P. umbellatus* is a source of important traditional Chinese medicine and has been used as diuretic for 2,500 years, with many other bioactivities reported, such as* in vivo* anticancer activity [3, 6]. However, only 264 EST sequences containing 3 SSR sites were described prior to September 2013.

Flanking sequences of SSR sites tend to be conserved, which allows the transfer of SSR primers between species. This transferability can be applied not only on close species, but also on distantly related ones. For an example, primers designed from EST-SSR sites of* Liriodendron chinense* could also amplify PCR products using* Michelia alba* DNA template [29]. The EST-SSR primers of* Pisum sativum* also could be applied on* Vicia faba* with transfer percentage of 60.74% [30], and EST-SSR primers designed from wheat efficiently amplified bands from DNAs of bean, maize, and rice [31]. Liao et al. [32] confirmed that EST-SSR primers of kiwi could transfer to apply on citrus fruits. The EST-SSR primers designed from cotton also could yield PCR products when using banana DNA template [33]. Our results also confirmed this primer transferability among families with transferability rate of 71% (Table 9). The development of EST-SSR primers was efficient, easy to operate with less cost, when compared with traditional molecular markers. More importantly, they are directly correlated with functional gene sequences, occupying higher information content. Therefore, it is very helpful for us to develop EST-SSR primers and then to confirm their transferability among species, especially when we want to know some genetic information about a species but it is lacking.

## 4. Experimental Section

### 4.1. Obtaining EST Sequences and Screening

All EST sequences from the order of Polyporales were downloaded from dbEST/GenBank (http://www.ncbi.nlm.nih.gov/dbEST/) as FASTA format. To remove those sequences with low quality or redundant sequences, a Perl script, EST-trimmer (http://pgrc.ipk-gatersleben.de/misa/), was used to remove poly A and poly T tails and those sequences with length shorter than 100 bp. The repetitive sequences were removed by using RepeatMasker software (http://www.repeatmasker.org/). The CD-HIT program (http://www.bioinformatics.org/cd-hit/) was employed to further remove redundant sequences through cluster analysis.

### 4.2. Searching of EST-SSR Markers

The Perl script, MISA (http://pgrc.ipk-gatersleben.de/misa/), was used to search EST-derived SSR markers with sequence longer than 18 bp. The number of repeats for mono-, di-, tri-, tetra-, penta-, and hexanucleotide motifs was at least 10, 6, 5, 5, and 5, respectively.

### 4.3. Functional Analysis of EST-SSRs in Ganodermataceae

Blast2GO was used to annotate gene function according to Gene Ontology (GO) categories [34]. Through sequence comparison in BLASTX, those EST-SSR sequences were compared with NR, Swiss-Port, and InterProScan protein databases under the condition of *E*-value less than 10^−5^. The GO terms were then summarized by WEGO software for EST-SSR sequences from the Ganodermataceae [35].

### 4.4. DNA Extraction

Thirteen natural sclerotium strains of* P. umbellatus* were collected from seven provinces in China, and voucher specimens were deposited at Northwest A&F University (Table 10). After activation culture on PDA (potato dextrose agar) medium [36], they were identified by mycelial growth, color of mycelium colony, liquid culture, polysaccharide content, and PCR amplification. Total genomic DNA was extracted from 100 mg fresh mycelium by using our improved cetyltrimethylammonium bromide (CTAB) method [37].

### 4.5. Primer Design and Screening

The Oligo 7 primer analysis software [38] was adopted to design primers from those EST sequences that contained SSR sites. The primers were designed upstream and downstream of SSR sites with sequence length ranging from 18 to 22 bp. The GC content in the primer ranged from 40 to 60% with annealing temperature about 60°C. Primers containing dimmers or hairpin structures were removed. Based on these criteria, primers could be designed only from those EST sequences in both Ganodermataceae and Corticiaceae. Thirty pairs of primers with different repeat motifs were synthesized by Sangon Biotech. Co. Ltd. (Shanghai, China) (Table 9). Based on the Tm value provided by the factory (Sangon), five gradient annealing temperatures (50, 52, 54, 56, 58, 60, and 62°C) were designed to screen the best value.

### 4.6. PCR Amplification

PCR amplifications were performed in a 25 *μ*L volume (50–80 ng DNA template, 0.2 *μ*M primers, and Taq-mix manufactured by Kangwei Co. Ltd., Beijing). The thermocycling parameters consisted of an initial denature step of 4 min at 94°C followed by 35 cycles of 94°C for 30 s, the highest annealing temperature 30 s, and 72°C for 45 s and a final extension step at 72°C for 7 min. The PCR products were separated on denaturing polyacrylamide gel (5%) and visualized after silver staining.

### 4.7. Genetic Diversity and Differentiation Analysis

Twenty one primer pairs showed clear and polymorphic bands, which was characterized by using 13* P. umbellatus* strains. Band positions for each mycelium and primer combination were scored as being either present (1) or absent (0). The scores were entered into a database program (Microsoft Excel) and compiled in a binary matrix. The number of alleles observed and expected heterozygosities were calculated to estimate genetic variation level by using POPGENE (version 1.31) [39].

The SIMQUAL program in NTSYS-pc System version 2.1 [40] was used to calculate the Jaccard similarity coefficient, and the dendrogram of genetic relatedness among 13 strains was produced by using the unweighted pair group method with arithmetic mean (UPGMA) analysis. The binary data for 13 strains were subjected to principal coordinate analysis (PCA) [41], and the first two principal coordinates were plotted to indicate the multilateral genetic relationships among them.

## 5. Conclusion

EST-SSR markers were developed from nonredundant EST sequences of Polyporales, in which the mononucleotide was the most abundant type and A/T was the predominant motif. There were 89.69% of EST-SSR markers that had nucleotide repetition more than 10 times. In five families of Polyporales, Ganodermataceae occupied the most EST-SSR markers, followed by Coriolaceae. The function of SSR marker-containing EST sequences in Ganodermataceae was separated into three groups, cellular component, biological process, and molecular function. Twenty one EST-SSR primers revealed polymorphism among natural 13* P. umbellatus* accessions with average PIC value 0.33 and transferability rate 71%. The 13 Chuling accessions showed relatively high genetic diversity. Both UPGMA dendrogram and principal coordinate analysis (PCA) showed the same cluster result that divided the 13 accessions into three or four groups. Our work provides informative satellite markers for future basic and applied research efforts related to* P. umbellatus*, including genetic diversity analysis for the resources in other countries, genetic linkage map construction, and molecular identification.

## Figures and Tables

**Table 1:** The information of EST-SSR markers in Polyporales.

Nucleotide repeat	Number	Of total (%)	Frequency	Number/Mb	Number of types	Predominant repeats	Number of predominant repeats
Mono-	8180	85.92	8.42	128.73	2	A/T	7200
Di-	260	2.73	0.27	4.09	4	AG/CT	141
Tri-	816	8.57	0.84	12.84	10	AGC/CTG	170
Tetra-	17	0.18	0.02	0.27	10	ACGC/CGTG	5
Penta-	94	0.99	0.09	1.48	5	AAAAT/ATTTT	79
Hexa-	153	1.61	0.16	2.41	24	ACCACG/CGTGGT	45
Total	9520	100	9.8	149.81	55	—	—

**Table 2:** Analysis of SSR markers in EST sequences of five families in Polyporales.

Nucleotide repeat	Ganodermataceae	Coriolaceae	Phanerochaetaceae	Polyporaceae	Total
Mono-					
Number	6530	1607	41	2	8180
Number /Mb	200	53	289	19	821
%	79.8	19.6	0.5	0.02	100
Di-					
Number	113	145	1	1	260
Number /Mb	3	5	7	10	25
%	43.46	55.77	0.38	0.38	100
Tri-					
Number	386	430	—	—	816
Number /Mb	12	14	—	—	26
%	47.3	52.7	—	—	100
Tetra-					
Number	8	9	—	—	17
Number /Mb	0.24	0.29	—	—	0.53
%	47.1	52.9	—	—	100
Penta-					
Number	82	12	—	—	94
Number /Mb	2.51	0.39	—	—	2.9
%	87.23	12.77	—	—	100
Hexa-					
Number	69	84	—	—	153
Number /Mb	2.1	2.7	—	—	4.8
%	45.1	54.9	—	—	100
Total					
Number	7188	2287	42	3	9520
Number /Mb	113.12	36	0.66	0.05	149.81
%	75.5	24.02	0.44	0.03	100

Notes: no SSR sites were found in the EST sequences of Meruliaceae.

**Table 3:** The sequence length of EST-SSR markers in five families of Polyporales.

Family	10–19 bp	20–29 bp	More than 30 bp	Total	Longest/bp
Ganodermataceae					
Number	3599	2403	1186	7188	164
%	50.07	33.43	16.50	100	
Coriolacae					
Number	1840	262	185	2287	126
%	80.45	11.46	8.09	100	
Phanerochaetaceae					
Number	21	13	8	42	31
%	50.00	30.95	19.05	100	
Polyporaceae					
Number	3	—	—	3	12
%	100	—	—	100	
Meruliaceae	—	—	—	—	—
Proportion %	57.38	28.13	14.49	1	—
Total	5463	2678	1379	9520	—

Notes: no SSR site was found in the EST sequences of Meruliaceae.

**Table 4:** Analysis of SSR markers in the EST sequences of Coriolaceae.

Nucleotide repeat	Number of SSR markers	Percentage (%)	Frequency %	Number of motif types	Abundant motifs	Number of abundant motifs	The percentage of abundant motifs (%)
Mono-	1607	70.27	3.33	2	C/G	853	53.08
Di-	145	6.34	0.30	4	AG/CT CG/CG	112	77.24
Tri-	430	18.80	0.89	10	AGC/CTG CCG/CGG	169	39.30
Tetra-	9	0.39	0.018	5	ACAT/ATGT ACGC/CGTG	6	66.67
Penta-	12	0.52	0.024	2	ATCGC/ATGCG	11	91.67
Hexa-	84	3.67	0.17	12	ACGAGC/CGTGCT AGCCTG/AGGCTC	71	84.52
Total	2287	100	4.74	35	—	1222	—

**Table 5:** Analysis of SSR markers in the EST sequences of Ganodermataceae.

Nucleotide repeat	Repeat motif type	Number of repeat motifs	Percentage (%)	Frequency of occurrence (%)	Average distance kb/site
Mono-	2	6530	90.8	13.51	5.00
Di-	4	113	1.57	0.23	289.09
Tri-	10	386	5.37	0.8	84.63
Tetra-	6	8	0.11	0.017	4083.37
Penta-	3	82	1.14	0.17	398.38
Hexa-	13	69	0.96	0.14	473.43
Total	38	7188	100	14.87	4.54

**Table 6:** The most abundant repeat motifs occurred in the EST-SSR sites of Ganodermataceae.

The most abundant repeat motif	Sequence length of repeat motif				Total	The longest motif (bp)
	≤9	10–19	20–29	≥30		
C	0	78	0	0	78	C (16)
G	0	40	0	0	40	G (18)
T	0	455	316	152	923	T (59)
CT	16	2	2	0	20	CT (35)
GA	12	2	3	17	34	GA (82)
TC	20	0	3	4	27	TC (44)
AAG	48	0	0	0	48	AAG (5)
ACC	11	0	0	0	11	ACC (9)
AGC	17	0	0	0	17	AGC (6)
CAC	13	0	0	0	13	CAC (6)
CAG	40	0	0	0	40	CAG (9)
CCA	10	0	0	0	10	CCA (6)
CGA	18	0	0	0	18	CGA (7)
CTC	10	1	0	0	11	CTC (10)
GCA	24	0	0	0	24	GCA (9)
GGC	14	0	0	0	14	GGC (6)
TCC	11	0	0	0	11	TCC (5)
TCG	23	0	0	0	23	TCG (6)
TTC	10	0	0	0	10	TTC (7)
TAAAA	79	0	0	0	79	TAAAA (7)
CACGAC	24	0	0	0	24	CACGAC (6)
GACCAC	4	16	0	0	20	GACCAC (10)

**Table 7:** Twenty-one EST-SSR primers selected out of 30 were evaluated for their amplifications on 13 *Polyporus umbellatus* accessions.

Primer code	Observed number of alleles (na)	Effective number of alleles (ne)	Polymorphism information content (PIC)	Observed heterozygosity (Ho)	Expected heterozygosity (He)	Nei's gene diversity	Shannon information index (*I*)
GL01	2	2.00	0.38	1.00	0.53	0.50	0.69
GL02	2	1.72	0.33	0.60	0.42	0.42	0.61
GL03	2	1.89	0.36	0.77	0.47	0.47	0.67
GL09	2	1.11	0.09	0.10	0.10	0.10	0.20
GL10	2	1.15	0.12	0.14	0.14	0.13	0.26
GL11	2	1.16	0.13	0.00	0.15	0.14	0.27
GL23	2	2.00	0.38	0.54	0.52	0.50	0.69
PS02	2	1.94	0.37	0.67	0.49	0.49	0.68
PS05	3	2.04	0.46	0.69	0.51	0.51	0.88
PS06	2	1.88	0.36	0.75	0.47	0.47	0.66
PS07	2	1.97	0.37	0.44	0.49	0.49	0.69
PS08	3	2.32	0.48	0.83	0.57	0.57	0.92
PS09	2	1.00	0.36	0.67	0.44	0.44	0.64
PS10	2	1.80	0.37	0.83	0.49	0.49	0.68
FP11	2	1.00	0.37	0.83	0.50	0.50	0.69
FP12	2	1.98	0.37	0.85	0.50	0.49	0.68
FP13	2	2.00	0.38	1.00	0.50	0.5	0.69
FP14	2	1.98	0.37	0.73	0.49	0.50	0.69
FP15	2	1.54	0.29	0.27	0.35	0.35	0.54
FP16	2	1.75	0.34	0.36	0.43	0.43	0.62
FP17	2	1.82	0.35	0.54	0.45	0.45	0.65
Mean	2	1.74	0.33	0.52	0.41	0.39	0.57

**Table 8:** Similarity coefficient of 13 strains of *Polyporus umbellatus* based on the amplification of 21 EST-SSR primers.

Strain code	1	2	3	4	5	6	9	10	11	12	13	14	16
1	1.00												
2	0.69	1.00											
3	0.76	0.56	1.00										
4	0.71	0.69	0.56	1.00									
5	0.80	0.67	0.69	0.64	1.00								
6	0.73	0.62	0.62	0.69	0.64	1.00							
9	0.62	0.58	0.60	0.51	0.64	0.62	1.00						
10	0.60	0.51	0.49	0.44	0.62	0.49	0.49	1.00					
11	0.60	0.42	0.58	0.44	0.58	0.49	0.58	0.78	1.00				
12	0.58	0.58	0.56	0.60	0.58	0.69	0.51	0.53	0.47	1.00			
13	0.58	0.62	0.47	0.73	0.58	0.69	0.42	0.44	0.42	0.60	1.00		
14	0.69	0.53	0.69	0.71	0.67	0.69	0.49	0.58	0.56	0.69	0.69	1.00	
16	0.60	0.53	0.56	0.62	0.62	0.64	0.58	0.60	0.51	0.76	0.58	0.79	1.00

**Table 9:** The 23 selected EST-SSR primers that could amplify PCR products by using *Polyporus umbellatus* DNA as template.

Primer^a^	Sequence (5′ to 3′)	GenBank access number	Tm value (°C)	Repeat motif
GL01	CTGCTCGTTGTACACGCTTC	HO719308.1	56	(GCA)7
	GAAAGACAGACGCGGGATTA			

GL02	GGTAATCTCGGCTTCACGAT	—	55	—
	TTGAGAATCTTCGGCACCTC			

GL03	AGTACGTAGCTGCCGTCCAG	—	58	—
	CCTGATACGTCCCGTAACCA			

GL09	GAGCTATCCAATCTATCGCCA	GO447546.1	55	(A)40⋯(A)29
	GATACCCCTGCTTTGAGTCC			

GL10	CTGACGACGTTAACCTAGGC	GO447482.1	53	(A)30⋯(A)32
	GCGTTGATACCCCTGCTAC			

GL11	ACCTCACTCTGTACTTATCCAC	GO447246.1	55	(G)10⋯(A)10
	CCCATTGGCCGAAAAGCTTA			

GL23	CGTCCCAGCAGCCGAGTCC	HO727333.1	63	(GCA)9⋯(CAG)9
	CCGCGACTCCAGGCCCGAA			

**PS01**	CTCGACGCATCAAATCACTCC	JK479811.1	55	(TC)5⋯(CCA)6
	ACCAGCTTGATCATGGACGAG			

PS02	GACGGGAAGGATCAGAACGAG	JK479807.1	55	(CAA)6
	GTCGACGAGTGAGATGAGGG			

PS05	CAACCGTCATCGATTTAACCC	JK479810.1	57	(ACT)6
	CCTCAAGATCTCCAGCGGTCA			

PS06	AAAGCATTCAAACAGCAGC	JK479808.1	55	(AAG)6
	GTACTGGCGTGTTGATGTTGG			

PS07	CAGCGGGCGTCTTTATTGTGT	JK479806.1	59	(CAA)6
	GTTGGTCCCCTTGCTGCCCTG			

PS08	CAAAGATCCAGCGTAACTCCC	JK479804.1	56	(TC)13
	CAACAGTGTAGTGACCACGAG			

PS09	TTCTTCACTCCATCCAGCCTT	JK479802.1	57	(CTA)6
	TTGGCGAGGGAGTAGTAGCTG			

PS10	ACTACTACTCCCACTACCACT	JK479802.1	57	(ACT)5
	GATGCGAATAAGATGGACAGT			

**FP10**	CTGGACACCCCGAACAACTGG	GR368360.1	58	(CAA)5
	CGACACCGCCTCAGTTGCCAT			

FP11	AGCGAGACTACAGCAGAGAC	GR369989.1	59	(GTC)6
	ACAGAGGACGTGGTGGAAGG			

FP12	TGCTCATCCACAACCGCCACC	GR370913.1	60	(CCA)6
	CTCCTTTCGGCAAGACCCAGT			

FP13	CACGTATCCCCATCCGCCAC	GR370248.1	60	(CG)7
	GAGGAGGGGCAGGACGAGGG			

FP14	GAACAAGACTGACCCCGCGTAT	GR370241.1	55	(CGA)5
	CACCCCAACAGGCAACTCTC			

FP15	CTGGACACCCCGAACAACTGG	GR370616.1	60	(CAA)5
	CGACACCGCCTCAGTTGCCAT			

FP16	GCCCCATCACATCGCGCTCT	GR368431.1	60	(CAA)5
	GTTGCCGCTGCCATACCCACT			

FP17	GCCGTCCGTGAAACCGTCCT	GR368155.1	60	(GAC)5
	GACAAGCGCCGCAGAGTCCAG			

^a^GL, PS, and FP represented that these primers were designed from EST-SSR sites of *Ganoderma lucidum*, *Puccinia striiformis* f. sp. *tritici*, and *Fomitopsis palustris*, respectively.

**Table 10:** Thirteen natural sclerotium strains of *Polyporus umbellatus* collected from China.

Strain code	Origin
1	Sunny slope of Taibai Mountain, Shaanxi Province
2	Shady slope of Taibai Mountain, Shaanxi Province
3	Shennongjia Mountain, Hubei Province
4	Jining, Shandong Province
5	Changbai Mountain, Jilin Province
6	Baishi Mountain, Hebei Province
9	Lueyang, Shaanxi Province
10	Fengxian, Shaanxi Province
11	Funiu Mountain, Henan Province
12	Changbai Mountain, Jilin Province
13	Daba Mountain, Sichuan Province
14	Jizu Mountain, Yunnan Province
16	Shennongjia, Hubei Province
